# Bimaxillary orthognathic surgery and condylectomy for mandibular condyle osteochondroma: a case report

**DOI:** 10.1186/s40902-015-0005-5

**Published:** 2015-02-05

**Authors:** Young-Wook Park, Woo-Young Lee, Kwang-Jun Kwon, Seong-Gon Kim, Suk-Keun Lee

**Affiliations:** 1 Department of Oral and Maxillofacial Surgery, College of Dentistry, Gangneung-Wonju National University, 7 jukheon-gil, Gangneung, 210-702 Gangwondo Republic of Korea; 2 Department of Oral Pathology, College of Dentistry, Gangneung-Wonju National University, Gangneung, Korea

**Keywords:** Osteochondroma, Condylectomy, Bimaxillary orthognathic surgery, BMP-4 expression

## Abstract

Osteochondroma is rarely reported in the maxillofacial region; however, it is prevalent in the mandibular condyle. This slowly growing tumor may lead to malocclusion and facial asymmetry. A 39-year-old woman complained of gradual development of anterior and posterior unilateral crossbite, which resulted in facial asymmetry. A radiological study disclosed a large tumor mass on the top of the left mandibular condyle. This bony tumor was surgically removed through condylectomy and the remaining condyle head was secured. Subsequently, bimaxillary orthognathic surgery was performed to correct facial asymmetry and malocclusion. Pathological diagnosis was osteochondroma; immunohistochemistry showed that the tumor exhibited a conspicuous expression of BMP-4 and BMP-2 but rarely expression of PCNA. There was no recurrence at least for 1 year after the operation. Patient’s functional and esthetic rehabilitation was uneventful.

## Background

Osteochondroma is one of the most common benign bone tumors (~40% of all benign tumors; 10% of all primary bone tumors) [1]. It usually occurs in the femur or tibia [2]. However, this tumor is rarely found in the maxillofacial region. The condyle and coronoid process of the mandible are the most prevalent sites of osteochondroma occurrence; however, relatively high incidence was also reported in the mandibular condyle [3].

Many options can be considered for the treatment of osteochondroma, including resection via local excision (condylectomy), arthroplasty, and vertical ramus osteotomy. Reconstruction with an autogenous bone graft such as costochondral graft or total joint replacement with a Temporomandibular joint prosthesis can also be good treatment options [1].

If the patient has malocclusion, two-step approaches, such as resection followed by orthognathic surgery have been used. However, there are few reports of mass resection with simultaneous orthognathic surgery. Here, we describe a case of mandibular condyle osteochondroma treated with bimaxillary orthognathic surgery as well as condylectomy.

## Case presentation

A 39-year-old woman was referred for facial asymmetry and malocclusion, which had slowly progressed over 4 years. She visited a dental hospital 2 years before admission and was diagnosed with chondroma by radiological observation. She did not experience any systemic diseases or accidental trauma. Although she had been treated for malocclusion in a local clinic, her malocclusion was not appropriately corrected but gradually worsened. Written informed consent was obtained from the patient for the publication of this report and any accompanying images.

**Figure 1:**
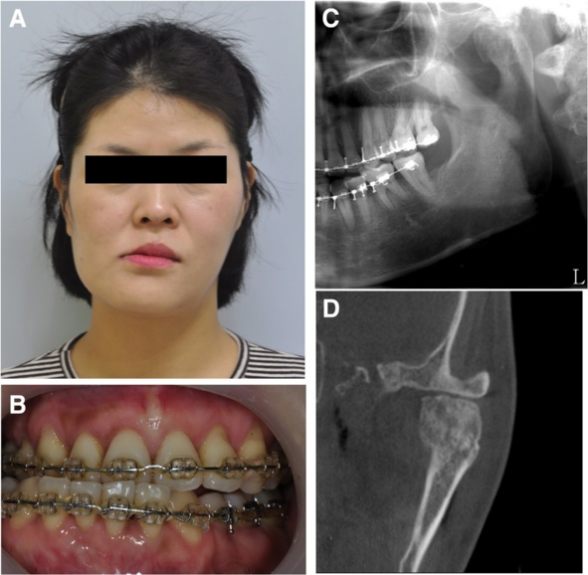
**Pre-operative patient information. (A)** The mandible was deviated to the right side in a clinical photograph. **(B)** The midline of the lower teeth was also deviated to the right side. **(C)** Panoramic radiograph shows the hypertrophic left mandibular condyle. **(D)** Cone-beam computed tomography image. A bony mass of irregular shape is detectable in the condyle.

Clinical examination revealed severe malocclusion and facial asymmetry. Intraorally, her midline of the mandibular teeth was deviated to the right side by up to 12 mm (Figures 1; A, B). She showed severe anterior crossbite and posterior crossbite on the right side, and an Angle Class III molar key on the left side and Class II molar key on the right side. She also complained of slight pain in her left TMJ during mouth opening. Her mouth opening was greatly shifted to the right side and was up to 35 mm.

Panoramic view revealed an irregular articular surface of the left mandibular condyle and a large bony mass (18 × 20 × 22 mm), which was diagnosed as probable osteochondroma. In cone-beam computed tomography (CBCT), bone marrow and a bony trabecular pattern were observed (Figures 1; C, D). A bone scan image showed a hot spot on the left mandibular condyle. Based on clinical and radiographic examination, the lesion of the left mandibular condyle was considered as osteochondroma.

**Figure 2:**
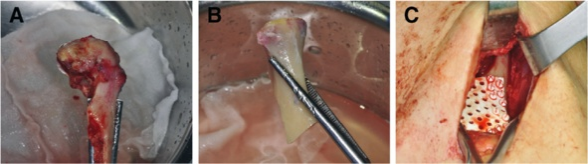
**Operation photographs. (A)** A condyle segment was taken out of the fossa. **(B)** Extraorally, high condylectomy was performed. **(C)** The segment was fixed via an absorbable mesh and screws.

Since the patient’s chief complains were facial asymmetry and malocclusion, condylectomy on the left condyle with simultaneous bimaxillary orthognathic surgery was planned. Because of the tumor growth, the patient’s maxillary dental arch was canted right and up by 3 mm in cephalometric analysis. Under general anesthesia, Le Fort I osteotomy was performed to correct maxillary canting. Sagittal split ramus osteotomy was also performed on the right side of the mandible and vertical ramus osteotomy was performed on the left side by a submandibular approach. After taking out a condylar segment, the irregularly out-growing tumor was removed along with a portion of normal tissue and condylectomy was also performed. To level both TMJs, a 5-mm fragment of cortical bone was harvested from the exposed mandibular angle area and grafted below the osteotomized segment (Figure 2). Then, the segment was reinserted into the condylar fossa. The distal segment of the mandible was positioned as guided by the final splint, and both sides of the mandible were fixed with an absorbable mesh and screws (Osteotrans-MX**®**; Takiron, Osaka, Japan).

**Figure 3:**
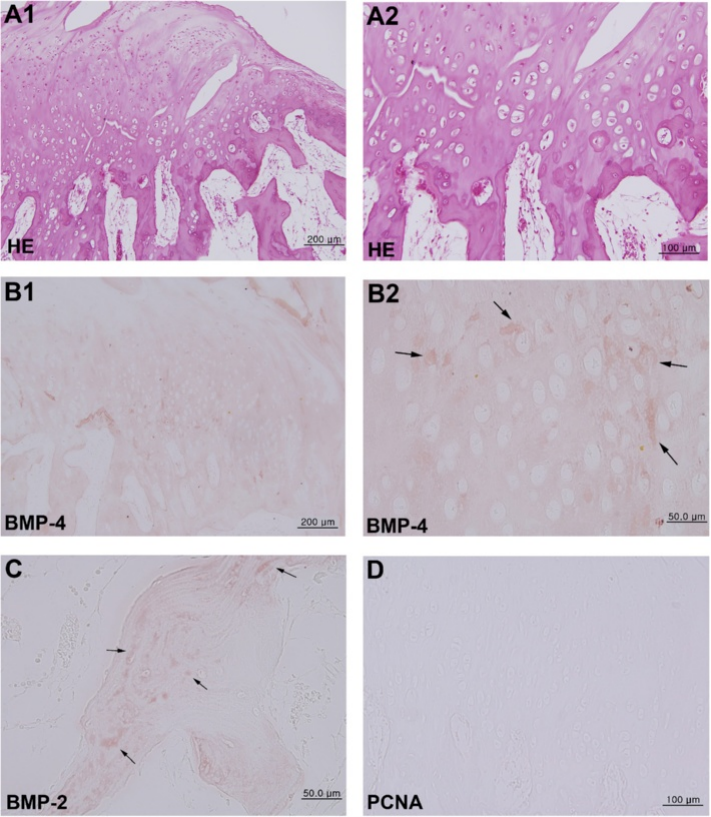
**Photomicrographs of osteochondroma. (A)** Hematoxylin and eosin staining showing proliferation of chondroid tissue deeply into marrow spaces, producing trabecular ossification. A2 is a higher magnification of panel A1. **(B)** Immunostaining for BMP-4, which is diffusely positive in the chondrocytes and surrounding matrix (arrows). **(C)** Immunostaining for BMP-2, which is slightly positive in the trabecular bone (arrows). **(D)** Immunostaining for PCNA, which is positive in a small number of tumor cells.

On histological examination, a chondroid mass was found on the capsule of the mandibular condyle, which was extended into underlying trabecular bones. The chondroid tissue showed many hyperplastic chondrocytes, which were mostly surrounded by hyalinized matrix and subsequently underwent ossification to produce trabecular bones. The underlying trabecular bones were irregular in shape and anastomosed each other, resulting in cancellous bone with abundant marrow stromal tissue (Figures 3; A1, A2). On immunohistochemical staining, the chondroid tissue was conspicuously positive for BMP-4 (bone morphogenetic protein-4; antibody was from Santa Cruz Biotechnology, Santa Cruz, CA, USA) (Figures 3; B1, B2) and the trabecular bones were slightly positive for BMP-2 (bone morphogenetic protein-2; antibody was from Santa Cruz Biotechnology) (Figure 3; C). This tumor was finally diagnosed as osteochondroma, and the entire tumor tissue examined was rarely positive for PCNA (proliferating cell nuclear antigen; antibody was from DAKO, Glostrup, Denmark) (Figure 3; D).

**Figure 4:**
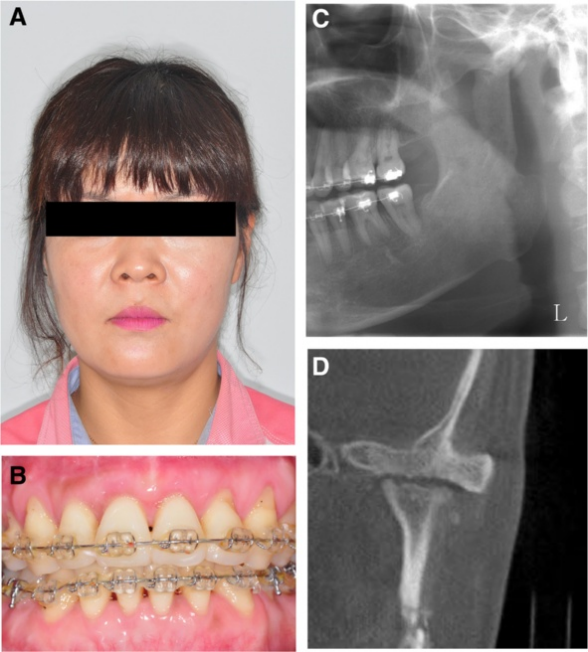
**Post-operative patient information. (A)** The patient showed no facial asymmetry. **(B)** The midline of the lower teeth was corrected. **(C)** Panoramic radiograph shows no signs of recurrence. **(D)** Cone-beam computed tomography image. The condyle was in a bone remodeling state.

**Figure 5:**
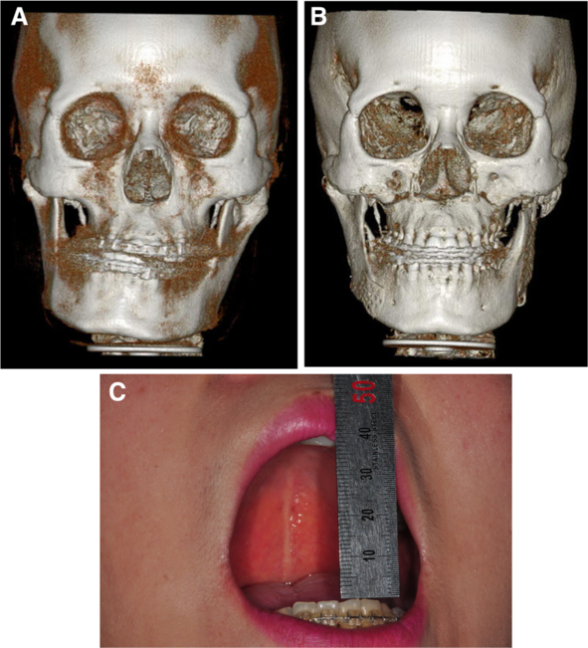
**Comparison of the pre-operation and post-operation status. (A)** A pre-operation 3D reconstruction. The mandible was deviated to the right side. **(B)** A 1-year post-operation 3D reconstruction. There were no deviation and no signs of recurrence. **(C)** The post-operation maximum mouth opening was 40 mm without pain or any interference.

The post-operative course was uneventful. Intermaxillary fixation was performed to stabilize the jaws for 2 weeks. The patient performed jaw movement exercises for 3 months after removal of intermaxillary fixation. At 12 months post-operation, the range of maximum mouth opening was 40 mm without pain or any interference, and no signs of recurrence were observed (Figures 4, 5).

## Discussion

Osteochondroma is a benign tumor of bone and cartilage. It is common in axial skeleton bones such as femur or tibia, but rare in the maxillofacial region [4]. In the mandibular condyle, osteochondroma may co-occur with chondroma and osteoma [3]. It occurs slightly more often in females than in males (1.22:1) [5]. Clinical manifestations of osteochondroma of the mandibular condyle are facial asymmetry, malocclusion, and joint pain [6]. Seki et al. reported a case of an osteochondroma patient with complete hearing loss [7]. Initially osteochondroma usually presents no symptoms, but symptoms may develop as the tumor size increases. The tumor may impinge on adjacent anatomic structures, such as nerves and bones [8]. Usually, slowly growing osteochondroma causes gradual vertical elongation of the affected side [6], but sometimes patients cannot recognize pathologic changes of their jaws. Our patient had facial asymmetry with chin deviation to the right side, severe malocclusion with crossbite and left TMJ pain.

A genetic component may be involved in this disease [9]. Another factor that increases the risk of osteochondroma is trauma [10]. However, the etiology of this disease is not clear. Our patient stated that she had no history of trauma to the left TMJ area and no systemic diseases.

Some patients exhibit vertical elongation of the affected side and slight pain [5]. Panoramic view and computed tomography (CT) can be valuable tools to diagnose this tumor. In panoramic view, osteochondroma can be detectable as an exophytic mass with mixed density and sclerosed appearance [1]. CT is more useful than panoramic view to visualize the mass and the relationships among adjacent anatomic structures [6].

Facial asymmetry and malocclusion may also be observed in condylar hyperplasia and other differential diagnoses such as osteoma, chondroma, fibrous dysplasia, fibrosarcoma, and chondrosarcoma [11]. Thus, histopathological diagnosis is important. Histopathologically, osteochondroma represents bone proliferation with a hyaline cartilage–capped osseous growth [2,3]. In contrast to other bone tumors, chondrocytes of osteochondroma show intracytoplasmic eosinophilic inclusions or hyaline globules inside them [2].

On histological observation, the present osteochondroma showed diffuse proliferation of chondroid tissue, which partly produced ossifying trabecular bones. The chondroid tissue was conspicuously positive for BMP-4 and the trabecular bones were slightly positive for BMP-2. Most chondrocytes were surrounded by hyalinized chondroid material and showed rare PCNA immunoreaction. Therefore, we presume that the present tumor was derived from condyle chondrocytes that showed ossification, and are confident in the osteochondroma diagnosis. We also believe that the present osteochondroma was a relatively well-differentiated benign tumor with low proliferative potential.

The protocol for treatment of osteochondroma of the mandibular condyle is controversial. If only the head of the condyle is involved without tumor extension into the neck, local resection or conservative condylectomy with contouring the affected condylar head can be the appropriate choice [1]. However, conservative approach may result in recurrence of the lesion or malignant changes [5]. In case of osteochondroma requiring the removal of the condylar head and neck, total condylectomy with joint reconstruction is recommended [12]. Costochondral or sternoclavicular grafts are considered for the reconstruction of the condyle, but in this case donor site morbidity and bone resorption are possible [13]. Alloplastic TMJ replacement may be performed, but it may lead to infection and heterotopic bone formation [14]. We performed high condylectomy to remove the mass. For 12 months after surgery, the patient had not complained of any discomfort and we could not find any signs of recurrence or malignant changes.

Deviation of the mandible because of osteochondroma of the mandibular condyle can also change the occlusion plane. In this case, orthognathic surgery should be considered. It can re-establish optimal occlusion and improve facial aesthetics [3]. There are many benefits of simultaneous TMJ and orthognathic surgery. First, only one operation under general anesthesia is required. Second, the surgeon can balance the occlusion, TMJs, jaws, and neuromuscular structure at the same time. It also reduces the overall treatment time [15]. In our case, the patient showed canting of the occlusion plane right and up by 3 mm. Because the chief complaints of the patient were facial asymmetry and malocclusion, we used both Le Fort I osteotomy and mandibular set-back surgery with removal of osteochondroma via condylectomy. It was possible that a small portion of the osteochondroma lesion still remained in the mandibular condyle. However, the patient’s mandibular condyle healed uneventfully and functioned well with no evidence of recurrence. Therefore, condylectomy performed in this study seemed to be appropriate. Although we are planning to perform additional follow-up checks, we presume that condylectomy with bimaxillary orthognathic surgery was effective in this patient. At 12 months post-operation, the patient was satisfied with the outcome.

## Conclusion

Osteochondromas of the mandibular condyle can represent facial asymmetry, malocclusion, and TMJ pain. It can also occur occlusion plane disharmony between maxilla and mandible. Bimaxillary orthognathic surgery is able to improve the occlusion status as well as the esthetic facial profile, simultaneously. The condylectomy with orthognathic surgery can be good choice to treat osteochondroma of mandibular condyle in facial deformity patients.
